# Seroprevalence of *Toxoplasma gondii* and *Salmonella* in Hunted Wild Boars from Two Different Regions in Switzerland

**DOI:** 10.3390/ani11082227

**Published:** 2021-07-29

**Authors:** Alessia Maria Giuseppina Bassi, Janine Carmen Steiner, Roger Stephan, Magdalena Nüesch-Inderbinen

**Affiliations:** Institute for Food Safety and Hygiene, Vetsuisse Faculty, University of Zurich, 8057 Zurich, Switzerland; alessibas@gmail.com (A.M.G.B.); janinecarmen.steiner@uzh.ch (J.C.S.); roger.stephan@uzh.ch (R.S.)

**Keywords:** *Toxoplasma gondii*, *Salmonella*, seroprevalence, wild boars, food safety

## Abstract

**Simple Summary:**

Wild boars are widely distributed in the northern and southern wooded regions of Switzerland and are popular for their meat. Wild boars can carry a variety of parasites, bacteria, and viruses that could infect humans and domestic animals. In this study, we focused on two important pathogens, the parasite *Toxoplasma gondii* and the bacterium *Salmonella*. We used enzyme-linked immunosorbent assay (ELISA) tests to detect antibodies to these pathogens in diaphragm tissue samples from hunted wild boars from two different regions of Switzerland. While the seroprevalence of *T. gondii* antibodies was similar for animals from the northern and southern region (29% and 37%, respectively), *Salmonella* seropositivity was very much higher in wild boars from the northern area (52%) than among animals from the south (5%). This may be related to the wild boar density, which may in turn be a risk factor for domestic animals and humans living in the same area. Pathogens in wild boars are of public health significance as a potential source of meat-borne diseases in humans.

**Abstract:**

*Toxoplasma gondii* and *Salmonella* are zoonotic foodborne pathogens that may be transmitted to humans through the consumption of raw or undercooked meat, including game. The aim of this study was to determine the seroprevalence of *T. gondii* and *Salmonella* antibodies in wild boars in two different regions in Switzerland. During the hunting season of 2020, a total of 126 diaphragm muscle samples of hunted wild boars were collected and the meat juice of these samples was analysed for pathogen-specific IgG antibodies using commercial enzyme-linked immunosorbent assay (ELISA) kits. The overall seroprevalences were 35% for *T. gondii* and 17% for *Salmonella*, respectively. In general, seropositivity increased with the age of the animals. Seroprevalences of *T. gondii* were similar for animals from the northern region (29%) to those from the southern region (36.8%), indicating that *T. gondii* is widespread in the sylvestrian environment. By contrast, *Salmonella* seropositivity was remarkably higher in wild boars from the north (52%) compared with those from the south (5.3%). The high occurrence of *Salmonella* may represent a risk of transmission to compatriot domestic animals such free-range farmed pigs as well as to humans. Further, meat of hunted wild boars may present a source of human toxoplasmosis or salmonellosis.

## 1. Introduction

The Eurasian wild boar (*Sus scrofa*) is widely distributed in most parts of Europe, including Switzerland [1]. In Switzerland, there are two separate wild boar populations consisting of a northern population ranging from Geneva to St. Gallen and a southern population distributed throughout the canton Ticino [1]. The two populations are separated by the Alps, which act as a natural physical and climatic barrier between northern and southern Switzerland. The past decades have seen an increase in the wild boar populations similar to what has been observed in other European countries [2].

Wild boars can serve as reservoirs for a range of pathogens, some of which are transmissible to domestic animals and humans, representing a potential veterinary and public health threat [3]. *Toxoplasma gondii* and *Salmonella* rank among the most important pathogens at the wildlife–livestock interface [4] and are listed as foodborne biological hazards related to game and game meat [5].

*T. gondii* is protozoan parasite that is able to infect warm-blooded animals, including livestock, wildlife, and humans [6,7]. In humans, *T. gondii* is transmitted by ingestion of raw or undercooked meat containing *T. gondii* tissue cysts or when handling contaminated material [8]. Domestic cats and other Felidae are the definitive hosts of *T. gondii* and, through faecal shedding, play a key role in the environmental dissemination of *T. gondii* oocysts [8]. Wild boars can serve as intermediate hosts upon ingestion of sources contaminated with sporulated oocysts. According to Rostami et al. [9], the estimated seroprevalence of *T. gondii* among wild boars in Europe is 26%. By contrast, in an earlier study from Switzerland, Berger-Schoch et al. reported a *T. gondii* seroprevalence of 6.7% among 150 analysed wild boars [10]. Therefore, current data on the seroprevalence of *T. gondii* among wild boars in Switzerland are required.

*Salmonella* spp. are gram-negative enteric bacteria and belong to the most important etiological agents of foodborne diarrheal diseases worldwide [11,12]. Wild boars may be carriers of various *Salmonella enterica* serovars, including *S. enteritidis* and *S. typhimurium*, which are most frequently implicated in human illness in Europe [5,13], and *S. cholerasuis*, which is a swine-adapted serovar infrequently reported among domestic pigs in Europe, but considered a major problem for the pig industry in North America and Asia [14]. Various studies estimate the seroprevalence of *Salmonella* among wild boar in Europe to be between 4% and 19% [2,15]. For Switzerland, an earlier study using cultural methods found that, in 2010, the prevalence of *Salmonella* in wild boars was 12% [16]. However, more recent data on the seroprevalence of *Salmonella* in wild boars are lacking. 

This study was designed to provide data on the seroprevalence of *T. gondii* and *Salmonella* antibodies in meat juice samples taken from wild boars shot during the hunting season of 2020.

## 2. Materials and Methods

### 2.1. Sampling 

Sampling took place during the hunting season (July to November) of 2020. All animals were legally hunted for human consumption. Diaphragm samples were made available post-mortem as part of the national compulsory inspection of wild boar for *Trichinella* spp., which is required by Swiss law. No animal was killed for the purpose of providing samples. Ethical approval was not required for this study.

**Figure 1:**
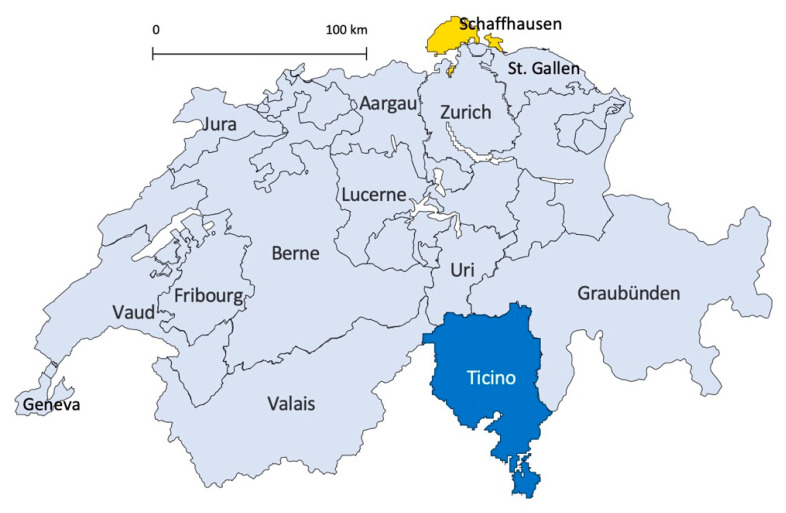
Map of Switzerland showing the cantons and the sampling areas. Wild boars belonging to group 1 originated from the canton Schaffhausen (indicated in yellow). Animals from group 2 were from the canton Ticino (shown in blue).

Samples originated from 31 wild boars shot in the canton Schaffhausen located in northern Switzerland (group 1) and from 95 wild boars shot in the canton Ticino located in southern Switzerland (group 2). Figure 1 shows the geographical location of the collection areas. Overall, 126 samples were available for analysis.

The weight and gender of the animals were determined by the hunters. In this study, the animals were divided into four weight categories: category I (10–20 kg), category II (21–40 kg), category III (41–60 kg), and category IV (>60 kg). For this study, the weight of each animal was taken as an approximation to its age. 

Samples were stored at −18 °C until processing. To obtain meat juice, the samples were thawed over night at room temperature and mechanically squeezed to obtain 200–500 µL of meat juice.

### 2.2. Detection of Toxoplasma gondii Antibodies

The samples were analysed using the PrioCHECK^®^ Toxoplasma Ab porcine Kit (Thermo Fisher Scientific, Prionics Lelystad B.V., Lelystad, The Netherlands). According to the manufacturer, the kit’s sensitivity and specificity is 97% and 100%, respectively, for porcine meat juice samples. Assays were carried out following the manufacturer’s instructions. In brief, 20 µL of meat juice was diluted with 180 µL of dilution buffer and 100 µL were transferred to the 96-microwell ELISA plate. After an incubation time of 60 min at room temperature, the plate was washed five times with 200 µL wash buffer and 100 µL conjugate was added. Conjugate incubation (30 min at 37 °C) followed by washing and the addition of 100 µL of chromogenic substrate. The substrate reaction was stopped after 15 min at room temperature, test plates were shaken manually for 10 s, and the reaction was read using a Synergy HTX Multi-Mode Microplate Reader (BioTek, Sursee, Switzerland) at 450 nm within 15 min. Test results were interpreted by calculating, for each sample, a percentage of positivity (PP) value according to the formula provided by the manufacturer. A PP ≥ 20 was regarded as positive and PP values below 20 were considered negative, as instructed by the manufacturer.

### 2.3. Detection of Salmonella Antibodies

The analysis for *Salmonella* antibodies was carried out using the PrioCHECK^®^ Porc. Salmonella Ab 2.0 Strip Kit for detection of antibodies against *Salmonella* LPS O-antigens 1, 4, 5, 6, 7, and 12 (Thermo Fisher Scientific, Prionics Lelystad B.V., Lelystad, The Netherlands), following the manufacturer’s instructions. This kit is the commercial version of the gold standard Salmonella mix-ELISA developed by the Danish Veterinary Institute (DVI). In short, 50 µL of meat juice was diluted with 100 µL of dilution buffer. After shaking for 2 min at 350 rpm, 20 µL of the samples was placed onto the 96-microwell ELISA plate. The plate was shaken for 2 min at 350 rpm followed by incubation for 30 min at room temperature. After incubation, the plate was washed four times with 200 µL wash buffer and 100 µL conjugate was added. Conjugate incubation (30 min at 37 °C) followed by another washing step and the addition of 100 µL of chromogenic substrate. The substrate reaction was stopped after 15 min at room temperature, and the reaction was read using a Synergy HTX Multi-Mode Microplate Reader (BioTek, Sursee, Switzerland) at 450 nm within 15 min. The test results were interpreted by calculating, for each sample, a PP value according to the formula provided by the manufacturer. A PP ≥ 40 was regarded as positive and PP values below 40 were considered negative, as instructed by the manufacturer.

### 2.4. Statistical Analysis

Seroprevalence differences were assessed using the two-tailed Fisher’s exact test and GraphPad Prism software (https://www.graphpad.com/QuickCalcs/, accessed on 21 June 2021). The results were deemed statistically significant if *p* < 0.05.

### 2.5. Geographical Maps

Geospatial visualization was carried out using Microsoft^®^ Excel 2020 version 16.43 powered by Bing ©GeoNames, Microsoft, TomTom.

## 3. Results

A total of 126 individual meat juice samples collected from wild boars shot during the hunting season from two different regions in Switzerland were analysed. 

Overall, 44 (35%) of the animals tested positive for the presence of antibodies against *T. gondii* (Table 1). The ELISA data revealed a slightly lower proportion of *T. gondii* seropositivity for animals belonging to group 1 (29%) compared with animals from group 2 (37%), but the difference was not significant (*p* = 0.518). The seropositivity of *T. gondii* was higher (56%) in animals belonging to weight category IV compared with animals belonging to categories I, II, and III (17%, 34%, and 31%, respectively); however, the difference was not quite statistically significant (*p* = 0.057).

The distribution of seropositivity for *T. gondii* among animals of different weight categories and genders is shown in Table 1.

**Figure 2:**
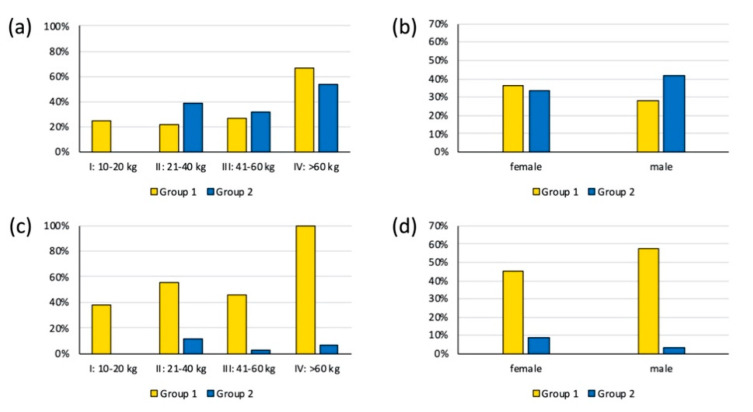
Seroprevalence of *Toxoplasma gondii* and *Salmonella* in hunted wild boars from two regions in Switzerland. Group 1: animals originating from the wild boar population in northern Switzerland; group 2: animals from southern Switzerland. (**a**) Percent of *T. gondii*-seropositive wild boars belonging to different weight categories; (**b**) percent of *T. gondii*-seropositive wild boars among female and male animals.; (**c**) percent of *Salmonella*-seropositive wild boars belonging to different weight categories; (**d**) percent of *Salmonella* seropositive wild boars among female and male animals.

Among the 31 animals of group 1, eight fell into weight category I (10–20 kg), nine were in category II (21–40 kg), 11 belonged to category III (41–60 kg), and three were assigned to category IV (>60 kg). Two (25%) of the animals in category I, two (22%) in category II, three (27%) in category III, and two (67%) in category IV were seropositive for *T. gondii* (Figure 2a). Further, in group 1, 11 were female, 18 were male, and the gender was not identified for 2 animals. With four (36%) of the females and five (28%) of the males testing positive, the seroprevalence for *T. gondii* was higher in females than in males (Figure 2b); however, the difference was not statistically significant (*p* = 0.694). Both animals of unidentified gender were seronegative.

Of the 95 wild boars in group 2, 4 belonged weight category I (10–20 kg), 26 were in category II (21–40 kg), 31 belonged to category III (41–60 kg), and 15 fell into category IV (>60 kg). Nineteen animals could not be assigned to any weight category. None of the animals in category I tested seropositive for *T. gondii.* Ten (38%) in category II, a further ten (32%) in category III, and eight (53%) in category IV were seropositive for *T. gondii* (Figure 2a). Seven (37%) of the animals of unknown weight also tested positive for *T. gondii*. Further, in group 2, 45 were female and 31 were male. For 19 animals, this information was not available. With 15 (33%) of the females and 13 (42%) of the males revealing positive test results, the *T. gondii* seroprevalence in female animals was slightly lower than in male animals (Figure 2b), and the difference was not significant (*p* = 0.477.

Overall, 21 (17%) of the animals tested positive for the presence of antibodies against *Salmonella* (Table 1). *Salmonella* seropositivity was significantly higher (*p* < 0.0001) for wild boars from group 1 (52%) compared with animals from group 2 (5%).

The distribution of seropositivity for *Salmonella* among wild boars of different weight categories and genders is shown in Table 1.

Of the 31 wild boars belonging to group 1, three (38%) in weight category I (10–20 kg), five (56%) in category II (21–40 kg), a further five (45%) in category III (41–60 kg), and all three (100%) belonging to category IV (>60 kg) were seropositive for *Salmonella* (Figure 2c). Further, in group 1, 5 (45%) of the females and 11 (58%) of the males tested positive for *Salmonella* (Figure 2d); however, the difference was not significant (*p* = 0.466). Both animals of unidentified gender were seronegative.

Among the 95 animals in group 2, none from weight category I were seropositive for *Salmonella*. Three (12%) from category II, one (3%) from category III, and one (7%) from category IV tested seropositive for *Salmonella* (Figure 2c). None of the animals of unknown weight tested positive. Further, in group 2, with four (9%) of the female and one (3%) of the male animals testing positive, the *Salmonella* seroprevalence among female animals was higher than among the male animals (Figure 2d), but the difference was not significant (*p* = 0.643).

Finally, seven (23%) animals in group 1 and two (2%) in group 2 were seropositive for both *T. gondii* and *Salmonella*.

## 4. Discussion

This study showed that wild boars in Switzerland are seropositive for *T. gondii* at an overall rate of 35%, which is remarkably higher than the prevalence of 6.7% reported in an earlier study from Switzerland [10], but comparable to pooled estimated *T. gondii* seroprevalences for wild boars in Europe (26%) as well as globally (23%) published in a systematic review and meta-analyses [9]. Previous studies have suggested an increase of seropositivity with age of the animal [10]. Taking the weight of each animal as an approximation to its age, we observed a higher seropositivity among the highest weight class; however, with our data, the correlation was not statistically significant.

In this study, the observed *T. gondii* seroprevalences were similar for animals originating from the wild boar population in northern Switzerland (group 1) and animals from southern Switzerland (group 2), suggesting that *T. gondii* is widespread in sylvestrian environments in both geographical areas. However, because investigations were limited to one canton for each population, no information is currently available on the distribution of these pathogens at the country level.

*T. gondii* infection in wild boars is asymptomatic and is not apparent as a pathological finding in hunted animals during meat inspection [17,18]. These factors emphasize the public health significance of *T. gondii* infection in wild boar as a potential source of meat-borne toxoplasmosis in humans and the importance of raising awareness among hunters and consumers alike. Persons at risk, such as immunocompromised people and pregnant women, should avoid ingestion of raw or undercooked wild boar meat [8].

The present study included an assessment of the prevalence of *Salmonella* antibodies among hunted wild boars in Switzerland. Our data revealed an overall *Salmonella*-seroprevalence of 17%, which is higher than that reported in recent studies from Italy (4%–6%) [13,19], but similar to the seroprevalence observed in Spain (19%) [15]. The overall observed increase of seropositivity with the age of the animal suggests that the animals act as reservoirs for *Salmonella* and are likely carriers and shedders. Notably, at group level, our study detected a significant difference in the proportion of *Salmonella* seropositive animals from group 1 (52%) and from group 2 (5%). The high *Salmonella* seroprevalence detected among wild boars from the northern region of Switzerland (canton Schaffhausen) was unexpected considering that *Salmonella* seropositivity in European wild boars is considerably lower. The possible reasons for the high number of animals infected with *Salmonella* in this region may include higher contact with contaminated agricultural areas and farmed animals. Differences in agricultural practices include a high proportion of arable land and poultry flocks in the canton Schaffhausen, whereas the proportion of agricultural land and farmed animals is lower in the canton Ticino [20]. However, additional investigations that include further risk factors are needed to explain our observations. This area of high seroprevalence among the wild boar population could play an important role in transmission of the pathogen to humans, livestock, and wildlife. This particular area also represents an area of high density of pig farms, thus the spatial overlap of roaming wild boar and outdoor pig farms may pose a potential risk of transmission events [1,21].

Further studies would be required to identify the *Salmonella* serotypes circulating among the wild boar population, in order to better assess the risk to human and animal health.

## 5. Conclusions

This study underlines the possible role of wild boars in the dissemination of *T. gondii* and *Salmonella* spp. The high occurrence of *Salmonella* spp. in wild boars may represent a risk factor for domestic animals and humans living in the same area. The data from this study may be useful for risk assessment on the consumption of meat and meat products of hunted wild boars.

## Figures and Tables

**Table 1:** Detection rates of *Toxoplasma gondii* and *Salmonella* antibodies (Abs) among meat juice samples from 126 wild boars from two different regions in Switzerland, as well as by weight and gender.

			No. (%) Tested Positive by ELISA for	
Feature	Category	No. Animals	*T. gondii* Ab	*Salmonella* Ab
Region				
	North (group 1)	31	9 (29)	16 (52)
	South (group 2)	95	35 (37)	5 (5)
	Total	126	44 (35)	21 (17)
Weight				
	10–20 kg (I)	12	2 (17)	3 (25)
	21–40 kg (II)	35	12 (34)	8 (23)
	41–60 kg (III)	42	13 (31)	6 (14)
	>60 kg (IV)	18	10 (56)	4 (22)
	nd	19	7 (37)	0 (0)
Gender				
	Female	56	19 (34)	9 (16)
	Male	49	18 (37)	12 (24)
	nd	21	7 (33)	0 (0)

nd, not determined.

## Data Availability

Data supporting the reported results are available from the authors upon reasonable request.
